# Psychosocial Burden and Strains of Pedagogues—Using the Job Demands-Resources Theory to Predict Burnout, Job Satisfaction, General State of Health, and Life Satisfaction

**DOI:** 10.3390/ijerph18157921

**Published:** 2021-07-27

**Authors:** Marie Drüge, Sandra Schladitz, Markus Antonius Wirtz, Karin Schleider

**Affiliations:** 1 Department of Psychology, University of Zurich, 8050 Zurich, Switzerland; 2 Heidelberg University, 69117 Heidelberg, Germany; sandra.schladitz@heiquality.uni-heidelberg.de; 3 Department of Psychology, University of Education Freiburg, 79117 Freiburg, Germany; markus.wirtz@ph-freiburg.de

**Keywords:** burnout, pedagogues, Job Demands-Resources Theory, job satisfaction

## Abstract

The current study examines the Job Demands-Resources theory among pedagogical professionals. A total of 466 pedagogues (*n* = 227 teachers; *n* = 239 social workers) completed the Copenhagen Psychosocial Questionnaire online. After testing the questionnaire structure using confirmatory factor analysis, a JD-R-based prediction model to predict effects of strains on the outcome constructs of burnout, job satisfaction, general state of health, and life satisfaction was estimated. The results confirm the questionnaire structure (RMSEA= 0.038; CFI = 0.94) as well as the fit of the prediction model (RMSEA = 0.039; CFI = 0.93). The outcome constructs could be predicted by emotional demands, work–privacy conflict, role conflicts, influence at work, scope for decision making, and opportunities for development (0.41 ≤ R² ≤ 0.57). Especially for life satisfaction, a moderator analysis proved the differences between teachers and social workers in the structure of the prediction model. For teachers, quantitative demands and work–privacy conflict are predictive, and for social workers, role conflicts and burnout are predictive. The study offers starting points for job-related measures of prevention and intervention.

## 1. Introduction

Psychosocial stress in the workplace and its consequences (e.g., exhaustion, life satisfaction, job satisfaction) have increasingly become a matter of public interest in recent years. Pedagogical professions are especially affected by stress consequences [1,2]. Both teachers as well as social workers count among these pedagogical professions and are particularly characterized by working with people and thus by interactions that create occupational field-specific demands and resources. In order to contribute to identifying and classifying demands and resources in these jobs, but also to developing possibilities for prevention and intervention, different models such as the Job Demands-Resources (JD-R) model [3] have been developed in order to predict the stress consequences based especially on work characteristics and conditions [4,5]. As this model is one of the most widely used across multiple professional areas and has been consistently updated to incorporate new findings [6], it provides a solid basis for research into work-related stress. To date, however, there has been no application of a JD-R-based model to educational occupational fields across the board, so in the present study, the constructs of burnout, job satisfaction, general health, and life satisfaction are to be predicted in pedagogues from occupational requirements and individual as well as social resources.

### 1.1. The Job Demands-Resources Model

The Job Demands-Resources (JD-R) model [3,7] attempts to predict stress consequences based on certain parameters. It has been expanded to the so-called Job Demands-Resources theory in recent years in order to account for causal and reversed causal effects within the originally proposed model [6]. Its main assumption is that each occupation contains both specific job demands (e.g., emotional or quantitative demands) and specific job resources (e.g., opportunities for growth or social support). According to the model, job demands and job resources each impact motivation and stress as well as job satisfaction. The JD-R theory comprises two main processes: The first process describes the relationship between demands, burnout, and health status (energetical process). The second process includes the connection between the acquisition or maintenance of resources and the improvement of work motivation and commitment (motivational process). Alarcon [4] was able to confirm these assumptions of the JD-R theory in a meta-analysis comprising of 231 studies. Burnout, commitment, and job satisfaction could be predicted in various occupations. 

### 1.2. Stress in Social and Pedagogical Professions

According to the German classification of occupations, teachers and social workers belong to the professional group of health, social, teaching, and educating professions [8]. In the following, we use “pedagogues” as a term for social workers and teachers. These professions have in common that they are often accompanied by a high level of human contact, interactions, and emotions, through which certain demands (e.g., quantitative or emotional demands) and resources (e.g., sense of community or social support) arise across these occupational fields [9]. As three common factors of 18 burnout theories, Schaufeli and Enzmann [10] described (1) the high motivation of the employee, (2) certain working conditions where aims cannot be fulfilled, and (3) dysfunctional coping mechanisms. In the field of social and educational professions, Schaufeli [11] suggests an unbalanced social exchange as the main reason for burnout. To know the working conditions for social and pedagogical professions in particular is the prerequisite to deriving tailor-made interventions from them. So far, however, the stress research referred to in the following tends to focus on either the professional group of teachers or that of social workers. 

### 1.3. Psychosocial Stress and Strain among Teachers

Research into teachers’ stress has increased significantly over the past three decades. Various reviews and meta-analyses attest to the critical stress conditions within the teaching profession with unique occupational stressors [12]. Kyriacou [1] based his review on sources of stress (e.g., poor student behavior), personality factors (e.g., belief in internal control), and coping strategies. McCarthy [12] and colleagues analysed 18 studies and identified the following important constructs: teacher’s job satisfaction and occupational commitment, burnout symptoms, stress prevention resources, and challenging student demands. A baseline survey of approximately 54,000 teachers showed that increased work-related influencing factors (e.g., emotional demands), as well as person-related factors (work–privacy conflict) and increased negative outcomes (e.g., burnout) were present in the teaching profession compared to the COPSOQ database [13]. However, teachers also reported higher job impact and greater development opportunities. Baeriswyl and colleagues [14] demonstrated the fit of the Demand-Control-Resources Model with teachers and integrated the construct of presenteeism (showing up for work sick) into the model framework. In addition to the large body of empirical evidence on negative stress consequences, teachers exhibit comparatively high levels of job satisfaction in both cross-sectional and longitudinal data [15]. Hakanen and colleagues [5] examined the JD-R model and showed that the effect of high job demands on health status was mediated by burnout, providing evidence of the energetical process in teachers. The concept of emotional labour and the according coping strategies received special attention in research on the teaching profession because of the job’s highly interactive nature [16]. In line with research into other professional groups, it has been shown that faking emotions can have a detrimental effect on teachers’ wellbeing and expressing true emotions can have a positive effect. Meanwhile, various stress interventions for teachers have been evaluated [17], addressing different domains of stress experience: knowledge-based, behavioral, cognitive-behavioral, and mindfulness approaches.

### 1.4. Psychosocial Stress and Strain in Social Workers

Compared to teachers, there is considerably less research on psychosocial stress and strain in social pedagogues. Studies often focus on specific fields of social work, such as child and youth welfare [18]. Some reviews are available, however [2,19]. Lloyd and colleagues [2] found that especially tension between philosophy and work-related demands as well as organizational factors of the work environment can lead to burnout, while social support or supervision could have a protective effect. McFadden and colleagues [19] examined child protection social workers regarding burnout and differentiated between organizational factors (e.g., workload, social support, supervision, organizational climate, job satisfaction) and individual factors (e.g., training, coping, compassion fatigue, compassion satisfaction). Poulson [20] conducted a study of professionals in child and youth care, from which some person-related and work-related factors of psychosocial stress can be derived: 80% of the participants described the workload as high, increasing, or even making them ill due to, among other things, high time pressure or conflicts among the workforce. Kim and Stoner [21] showed that job autonomy interacted with role stress in predicting burnout, while social support interacted with role stress in predicting turnover intention in social workers. Nübling and colleagues [22] found high emotional demands and work–privacy conflict, but also high life satisfaction. Studies based on the JD-R model have not yet been conducted for this profession. Various interventions for stress reduction, burnout prevention, and wellbeing have also been evaluated in the field of social work, although comparatively fewer than in the occupational group of teachers. Especially mindfulness-based interventions showed promising results [23]. 

### 1.5. Research Objectives

The present study aims to examine assumptions within the JD-R theory in pedagogues; to predict burnout, job satisfaction, general health as well as life satisfaction; and to examine the occupation-specific differences in the predictive structure of the model. The following question arises: Can the JD-R theory be used as an adequate model in educational contexts to predict burnout, job satisfaction, general health, and life satisfaction in this context? Does the profession have an influence on the predictive structure? Accordingly, we test the following hypotheses.

**Hypothesis** **1** **(H1).** *Demands are positively related to negative outcomes (burnout, general health) and negatively related to positive outcomes (job satisfaction, life satisfaction).*

**Hypothesis** **2** **(H2).** *Resources are negatively related to negative outcomes and positively related to positive outcomes.*

**Hypothesis** **3** **(H3).** *The profession of teacher vs. social worker serves as a moderator in the predictive structure.*

## 2. Materials and Methods

### 2.1. Questionnaires

Psychosocial stress and strain were assessed online in a cross-sectional design using the Copenhagen Psychosocial Questionnaire (COPSOQ) [24] in its German short form, comprising 25 scales with 87 items [13]. Validation and psychometric testing of this German version was conducted in 2005 on 2561 employees. The items used are presented in 5-point Likert scales (e.g., ‘always’, ‘often’, ‘sometimes’, ‘rarely’, ‘never/almost never’). Graded point values (minimum 0, maximum = 100) can be assigned to the answers. The following COPSOQ scales were used to measure demands: quantitative demands (4 items, α = 0.78), emotional demands (3 items, α = 0.75), work–privacy conflict (5 items, α = 0.91), and role conflict (4 items, α = 0.78). The following scales represented resources: influence at work (4 items, α = 0.69), decision latitude (4 items, α = 0.90), opportunities for development (4 items, α = 0.73), social support (4 items, α = 0.83), sense of community (3 items, α = 0.82), and predictability (2 items, α = 0.74). Negative consequences of demands were burnout (6 items, α = 0.88) and general health (single item, ranging from worst to best conceivable state of health), and the positive consequences were job satisfaction (7 items, α = 0.80) and life satisfaction (5 items, α = 0.89). 

### 2.2. Sample

Three inclusion criteria were used to obtain the sample: 1. a completed university degree (diploma, bachelor’s degree, master’s degree, state examination) in the social field or in teaching, 2. working in a social work context according to [18] or in a school, and 3. in the German state of Baden-Wuerttemberg. In order to recruit the social workers, a list of 1200 employees in the region was first compiled through an online search, who were then asked to cooperate, of which, 239 data records could be included in the sample. To recruit the teachers, 33 schools of different school types in the region were randomly selected and contacted, reaching approximately 1200 teachers. After approval by the school management, attention was drawn to the study in the schools (e.g., teacher conferences). In total, 466 pedagogues participated in the study, namely 239 social workers and 227 teachers. Professional experience varied: 7.9% have been employed for less than one year, 28.9% for 1–4 years, 31.4% for 5–14 years, 20.5% for 15–24 years (20.5%), and 11.4% for 25 years or more. Furthermore, 66.5% (*n* = 159) of the social workers and 72.2% (*n* = 164) of the teachers were female. The social workers’ ages lay between 22 and 65 years (*M* = 42.5, *SD* = 11.6) and the teachers’ ages were between 24 and 65 years (*M* = 42.2; *SD* = 11.9). Social workers cited child and youth welfare (56.1%), social assistance (32.2%), health assistance (9.2%), and assistance for the elderly (2.5%) as their places of employment, while teachers were employed in different types of schools in the German educational system (multiple answers possible): elementary schools (23.4%), various types of secondary schools (86.8%), and vocational schools (1.2%). A total of 227 usable data sets were collected online in such a way that no missing values could occur.

### 2.3. Statistical Analyses

To test whether the constructs are interrelated, structural equation modelling presents an adequate method of analysis [25]. Since the procedure assumes multivariate normal distribution of the analyzed variables, these were previously tested and the criteria formulated by Kline [25] were proven to be met (skewness ≥ 3, kurtosis ≥ 8). The questionnaire structure was tested using confirmatory factor analysis (CFA). Model parameters were determined by maximum likelihood estimation. Global as well as local model fit indices were used to test the model fit. Global fit indices indicate congruence of the empirical variance–covariance matrix with the data structure predicted by the model. To assess global goodness of fit, the chi-squared test (χ²-test), the ratio of the χ²-value to the degrees of freedom (df), the Normed Fit Index (NFI), Tucker Lewis Index (TLI), Goodness of FIT Index (GFI), Comparative Fit Index (CFI), and Root-Mean-Square-Error of Approximation (RMSEA) are of importance [25]. High values in NFI, TLI, GFI, and CFI (≥0.90) [26] and a low RMSEA (≤0.06) [27] indicate a good model fit. Local fit measures indicate the goodness with which individual constructs are represented by their indicators. For this purpose, indicator reliability (≥0.40) [28] was used, as well as factor reliability (≥.60) [29] and average extracted variance (DEV ≥0.50) [30]. In a second modelling step, the model-based predictive relationships were inserted into the model and the significance of the postulated predictive weights, and their effect sizes assessed. Finally, a moderation analysis tested the prediction model in an occupational comparison (teachers and social work professionals). This was done by means of nested model comparisons in which it was tested whether a significantly worse data fit resulted from the group-invariant parameter specification [31].

## 3. Results

### 3.1. Confirmatory Factor Analysis of the JD-R Model

First, in a confirmatory factor analysis, we tested the item-construct fit (measurement models) on the one hand and the correlations of the outcome variables burnout, job satisfaction, general health, and life satisfaction in the overall model (structural model) on the other hand. The global goodness of fit (RMSEA = 0.047; CFI = 0.89; Table 1, row Original CFA) indicates that, according to the CFI, there are moderate limitations to the model fit. To identify systematic model violations, we examined indicator reliabilities (IRs) and violations of local stochastic independence using modification indices. Indicator reliabilities of individual items (in the scale role conflict RC_1: ‘Do you do things at work which are accepted by some people but not by others?’, IR = 0.21; in the scale job satisfaction JS_1: ‘Regarding your work in general: How pleased are you with your work prospects?’, IR = 0.24; JS_2: ‘… with the people you work with?’, JS = 0.34; JS_3: ‘… with the physical working conditions?’, IR = 0.25) indicated unacceptable indicator–construct associations (IR < 0.4). Furthermore, the item JS_4: ‘Regarding your work in general. How pleased are you with the way your department is run?’ showed high local dependencies with other individual items. This indicated an incompatibility of these individual items with the model, and accordingly, these items were removed. The items JS_5 ‘Regarding your work in general: How pleased are you with the way, your abilities are used?’, JS_6 ‘…interests and skills involved in your job?’, and JS_7 ‘… your job as a whole, taken everything into consideration?’ were used for Job Satisfaction. The reliability of the two scales was acceptable (for role conflict α = 0.79; for job satisfaction α = 0.79). Despite a significant residual correlation of the two burnout items (Burnout_1: ‘These questions are about how you have been during the last 4 weeks. How often have you felt worn out?’, Burnout_2: ‘How often have you been physically exhausted?’), they were retained in the modified model because of their high item–construct correlation. The construct social support proved to be two-dimensional and was broken down into the subcomponents social support vertically from colleagues (two items) and social support horizontally from a supervisor (two items).

The aforementioned model modifications substantially improved the initially moderate model fit to a good fit (RMSEA = 0.038; CFI = 0.94) while maintaining the basic model structure. The significance of the χ² statistic can be considered noncritical for an adequate model structure due to the large sample size [25]. Considering local fit, overall satisfactory values resulted for the items (indicator reliability) and the constructs (especially factor reliability > 0.60, Table 2). Although the quality of the measurement model of the factors’ influence at work proved critical, this construct was nevertheless included in the model due to the thematic weighting in the JD-R.

### 3.2. Predictive Model 

The predictive model based on the JD-R model included ten predictor constructs (Table 2), which were simultaneously included in the model as correlated constructs to predict stress consequences (burnout, general health, job satisfaction, and life satisfaction).

The analysis showed that according to the global (Table 1, row Pathway Model Model; RMSEA = 0.039, CFI = 0.93) and local goodness of fit indices, the data adequately represented the model. The intercorrelations of the predictor constructs are shown in Table 3. The divergent validity of the factor structure at the predictor level is confirmed by the fact that the construct intercorrelations of the relation are lower than the root of the average variance, which can be interpreted as an estimate of the average indicator–construct correlation [30].

**Figure 1:**
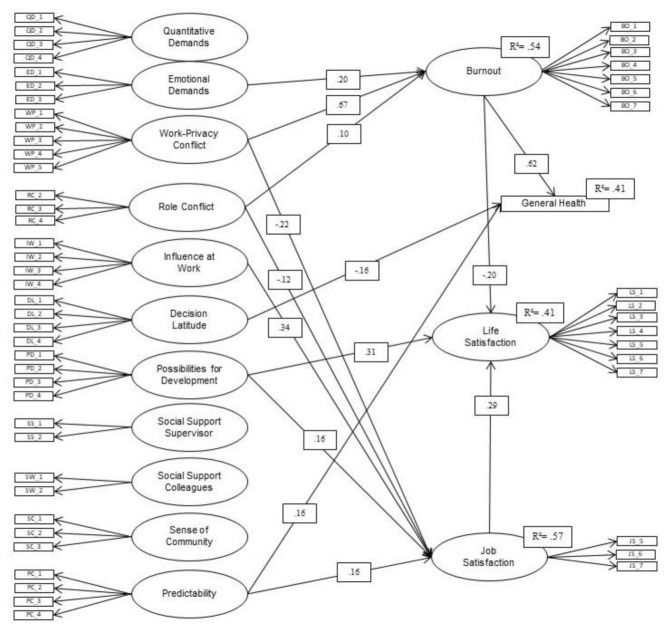
Predictive model.

The model structure can be seen in Figure 1: burnout can be predicted by 54% and job satisfaction by 57%. Life satisfaction and general health can be predicted by the predictors as well as the other stress consequences by 41% each. In the case of significant relationships, demands are positively associated with negative outcomes and negatively associated with positive outcomes (H1). Resources are negatively associated with negative outcomes and positively associated with positive outcomes (H2). The indirect path of demands and resources on burnout resulted in a stronger variance explanation of general health than the direct prediction of general health by demands and resources. Similarly, the indirect pathways of demands and resources on burnout and job satisfaction resulted in a higher explained variance of life satisfaction.

### 3.3. Moderator Analysis Based on Profession

The correlations of the JD-R prediction model were furthermore tested in a moderation analysis comparing the occupational groups teachers and social workers. A total of eight differences in the predictive structure emerged (see Table 4): for teachers, the positive predictive weights of emotional demands on burnout and those of quantitative demands on life satisfaction are significantly stronger. For social workers, these pathways did not prove significant, but for them, the predictive weights of predictability on job satisfaction and of role clarity on life satisfaction were stronger. For teachers, these paths were not significant. The relationship between influence at work and life satisfaction proved positive for teachers and negative for social workers. The negative effects of work–privacy conflict on life satisfaction and of influence at work on general health are both stronger among teachers and significant only in this group. The negative effect of burnout on life satisfaction is significant only among social work professionals. For teachers, a substantially higher proportion of variance in life satisfaction can be explained by the model at 55% (*R*^2^_SW_ = 44%).

Overall, it can be deduced from the moderator analysis that the occupation substantially moderates the above-mentioned correlations in the prediction model. Accordingly, H3 can be considered confirmed only for the described correlations. 

## 4. Discussion

We were able to use JD-R theory to predict burnout, job satisfaction, general health, and life satisfaction among pedagogues. 

### 4.1. Confirmatory Factor Analysis 

The theory-based modeling of the COPSOQ scales were shown to be compatible to the data in its basic structure after eliminating four items (RMSEA = 0.038; CFI = 0.94). Only for the factor of influence at work did moderate local fit problems continue to appear. Due to its high theoretical relevance [3], it was nevertheless left in the model. The question as to why this scale, which has been shown to be internally consistent in other studies and occupational groups, did not work should be the subject of further empirical testing. All in all, the COPSOQ scales were found to be suitable for empirical testing of the structure of the JD-R model on this basis.

### 4.2. Predictive Model for Strain 

For the structural model based on the JD-R model, which assumes that the demands and resources are essential predictors of strain, it was shown that burnout can be predicted by the factors of emotional demands, work–privacy conflict, and role conflict at 54%. The energetical process [5] between work demands and burnout could thus be confirmed. Interestingly, the stressors in the model are more important than the resources. This emphasizes the high importance of the motivational process compared to the buffering effect of the resources. Special attention is given to the factor of work–privacy conflict, which is why this should be specifically investigated in follow-up studies in order to develop and evaluate possible methods to prevent burnout in this area. Social support from colleagues and from superiors are considered important factors, especially in educational professions [19,21]. However, the buffers of influence at work and social support found in other studies are of lesser importance here, which is surprising as in previous reviews team support played a central role alongside supervision [2,21]. One reason for our finding could be the heterogeneous professional groups in the sample, as previous research (particularly in the field of social work) tended to focus on one professional field (e.g., child protection social workers) [19].

Job satisfaction can be predicted by work–privacy conflict, role conflict, influence at work, and development opportunities at 57%. Influence at work has the highest predictive value, which is in line with the JD-R theory [3,7] as well as previous research. Especially, role conflict and work–privacy conflict are explicitly found for social and educational professions [1,2,11,32]. Influence at work and development opportunities could also be confirmed by previous research [13].

General health can be predicted by the factor of decision-making latitude and the indirect path through burnout to a total of 41%. It could only be explained at 27% in total via the predictors, which speaks for the mediating role of burnout on general health. Mediator analyses for the connection in the energetical process [5] between job demands, burnout, and the general state of health, which also take into account occupational-field-specific aspects in the sense of potential moderation effects, offer an interesting perspective for a more differentiated understanding of the dependencies.

Life satisfaction can be predicted by the factor development opportunities and via indirect pathways by burnout and job satisfaction at 41%. Since the positive correlations between life satisfaction and job satisfaction have been demonstrated previously [33], this finding is not surprising. At the same time, life satisfaction is usually associated with other (private) factors in addition to work-related factors, so the 41% clarification can be seen as high. This could possibly be associated with the high importance of their work among pedagogues [1,2], which, on the one hand, could be seen as positive, since this could be linked to a high level of motivation (motivational process), but at the same time, could also represent a danger in the context of the work–privacy conflict. However, statements on this are to be understood speculatively and should be investigated further.

### 4.3. Moderator Analysis Based on Profession

Occupation-specific differences in the predictive structure emerged in the moderator analysis. The prediction of life satisfaction is very clearly moderated by occupation. The authors of [34] emphasize the difficulty of modelling a predictive model for life satisfaction. The more subpopulation-specific dependencies and effect processes are empirically differentially significant, the more cautiously cross-population findings must be interpreted. For teachers (*R*^2^_T_ = 0.55 vs. *R*^2^_SW_ = 0.44), the predictive power with regard to life satisfaction is significantly better overall. The relevance of the differential findings, for example, becomes clear when considering that burnout has been shown to be high in both professions [13,22]. However, the moderator analysis indicates that this must be considered more relevant for teachers—due to the significantly higher predictive weight—with regard to life satisfaction. There were two differences in the influence of work: one on life satisfaction and the other on health status. A possible explanation might be that the items measuring influence of work (e.g., influence on the quantity of the work) cannot be transferred without restrictions to the teaching occupation. This is also suggested by problems of construct assessment in the confirmatory factor analysis (critical factor and item reliabilities). In addition to these differences, however, there are many similarities in the predictive structure between the two pedagogical professions, especially in the areas that explain the energetical process of the JD-R model (demands-burnout-general health).

### 4.4. Limitations and Future Research

The presented results can only be interpreted with some limitations: The sample is not a representative sample—although it shows strong overlaps in terms of gender and age in pedagogical occupations compared to data from the Institute for Labor Market and Career Opportunities [35], men are slightly underrepresented in the sample of teachers. The opportunity sample was recruited only in Germany; this does not allow for generalizable conclusions, as working conditions in particular can vary due to the educational policies in different countries. In addition, the overall sample is heterogeneous with many different occupational groups, which is a weakness on the one hand, but also a strength on the other, as it allows for the similarities and differences between the social and educational professions to be identified, which has been neglected in previous research. Similarly, a low response rate of 20% was noted; this may have been due in part to the fact that attention was drawn to missing items, which may have led to dropout. The online format makes it difficult to access certain groups of participants and leads to non-standardized conditions during implementation. In the study, cross-sectional data were analyzed, so that the deduction of causal effects must be understood as an assumption based on the theoretical framework. This may be critical with regard to the status of the constructs of work–privacy conflict or role conflict since these could fully or partially be considered as a consequence of stress. In order to increase the evidence for causal relationships, interventional designs should be used in future research. In addition to these limitations, however, the results are of great importance for practice and research, especially with regard to the well-founded model orientation, the methodical soundness of the estimation of the model parameters, and as a basis for prevention and intervention purposes. Since pedagogical professions are affected by high psychosocial stress and strain [1,2,13], research on prevention and intervention options has already been a focus in the past [17,23]. However, work–privacy conflict, role conflict, and emotional stress on the side of demands and decision-making scope, development opportunities, and influence at work on the side of resources should be considered in order to develop appropriate measures and concepts and to empirically test them in specific occupational fields and adapted if necessary. Possible starting points could be the expansion of collegial counselling and external supervision, which could be linked to some of the requirements and resources mentioned.

## 5. Conclusions

Psychosocial stress in the workplace and its consequences (e.g., poor general health or life satisfaction) have increasingly become a matter of public interest in recent years. Special interest has been drawn to pedagogical and professions (teachers, social workers) due to a specific pattern of stress related to the social nature of the work. The theory-based model of the COPSOQ scales was shown to be compatible to the data, therefore, the outcomes of burnout, job satisfaction, general health, and life satisfaction were estimated through the predictors. For the structural model based on the JD-R model, which assumes demands and resources as essential predictors of strain, it was shown that burnout can be predicted by the factors of emotional demands, work–privacy conflict, and role conflict to 54%. Occupation-specific differences in the predictive structure emerged in the moderator analysis; especially for life satisfaction, a moderator analysis proved differences between teachers and social workers in the structure. For teachers, quantitative demands and work–privacy conflict are predictive, and for social workers, role conflicts and burnout are predictive. Therefore, this research could be used as a starting point for future research as well as for the development and evaluation of appropriate measures and interventions. 

## Figures and Tables

**Table 1:** Global fit indices (*n* = 466).

	χ²	Df	*p*	χ²/df	TLI	CFI	RMSEA
Threshold Acceptable Fit ^1^			<0.001	≤2.5	≥0.90	≥0.90	≤0.06
Original CFA	2755.06	1354	0.000	2.035	0.88	0.89	0.047
Modified CFA	1825.34	1086	0.000	1.681	0.93	0.94	0.038
Path Model	1929.31	1112	0.000	1.72	0.92	0.93	0.039

^1^ [25,26,27].

**Table 2:** COPSOQ scales and local goodness of fit indices of the structural model (*n* = 466).

Dimension and Item Content ^1^	IR ^2^	CR ^3^	FR ^4^	DEV ^5^
Thresholds	≥0.4		≥0.6	≥0.5
Quantitative Demands			0.79	0.48
QD_1 Working fast	0.34	11.19 ***		
QD_2 Too much work	0.52	13.53 ***		
QD_3 Not enough time	0.53	13.62 ***		
QD_4 Overtime	0.51	^a^		
Emotional Demands			0.76	0.52
ED_1 Emotionally stressful situations	0.36	11.41 ***		
ED_2 Emotionally involved	0.54	13.06 ***		
ED_3 Emotionally demanding	0.66	^a^		
Work-Privacy Conflict			0.91	0.68
WP_1 Private life disturbed by work	0.63	21.51 ***		
WP_2 Time commitment complicates private duties	0.78	25.67 ***		
WP_3 Private things are left undone	0.71	23.67 ***		
WP_4 Work generates private stress	0.75	^a^		
WP_5 private plans are changed	0.54	18.74 ***		
Role-Conflicts			0.80	0.57
RC_2 Contradictory demands	0.46	^a^		
RC_3 Contradictory accuracy	0.74	14.15 ***		
RC_4 Unnecessary demands	0.54	13.14 ***		
Influence at Work			0.69	0.35
IW_1 Influence on the work	0.48	10.06 ***		
IW_2 Influence on collaboration	0.27	8.46 ***		
IW_3 Influence on the amount of work	0.35	9.24 ***		
IW_4 Influence what is done	0.37	^a^		
Decision Latitude			0.90	0.69
DL_1 Breaks arrangement	0.65	20.46 ***		
DL_2 Vacation arrangement	0.66	20.81 ***		
DL_3 Work interrupt colleague conversations	0.70	21.58 ***		
DL_4 Do private things (during work)	0.73	^a^		
Possibilities for Development			0.72	0.42
PD_1 Variation of work	0.50	11.35 ***		
PD_2 Taking initiative	0.30	9.47 ***		
PD_3 Learning new things	0.48	11.24 ***		
PD_4 Apply skills/expertise	0.37	^a^		
Social Support by Colleagues			0.78	0.64
SW_1 Supporting Colleagues	0.50			
SW_2 Colleagues listen to work problems	0.58			
Social Support by Supervisor			0.84	0.72
SS_1 Supporting Supervisor	0.56			
SS_2 Supervisor listens to work problems	0.54			
Sense of Community			0.85	0.65
SC_1 Good working atmosphere	0.68	18.61 ***		
SC_2 Good cooperation	0.62	17.36 ***		
SC_3 Part of the community	0.65	^a^		
Predictability			0.73	0.58
PC_1 Receiving changes in advance	0.50			
PC_2 Receiving information needed	0.70			
Burnout			0.89	0.58
BO_1 Fatigue	0.40	^a^		
BO_2 Physical exhaustion	0.51	13.04 ***		
BO_3 Emotional exhaustion	0.56	13.46 ***		
BO_4 Not able to perform	0.66	14.34 ***		
BO_5 Feeling worn out	0.76	15.08 ***		
BO_6 Weak/disease prone	0.49	12.81 ***		
Job Satisfaction			0.79	56
JS_5 How own skills are used	0.59	^a^		
JS_6 Challenges and skills	0.43	13.89 ***		
JS_7 Work in general	0.54	15.74 ***		
General Health				
Life Satisfaction			0.89	0.61
LS_1 Life corresponds to ideal expectations	0.76	^a^		
LS_2 Excellent living conditions	0.64	21.03 ***		
LS_3 Being satisfied with life	0.71	22.91 ***		
LS_4 Dreams achieved in life	0.58	19.48 ***		
LS_5 Hardly any change requests	0.48	16.90 ***		

^1^ for detailed contents, see (Nübling, 2005); ^2^ indicator reliability; ^3^ critical ratio; ^4^ factor reliability; ^5^ thresholds, see Section 4.3; ^a^ unstandardized values were set equal to 1 to allow identifiability. *** *p* ≤ 0.001.

**Table 3:** Correlation of predictors (*n* = 466). The diagonal indicates the root of the average variance as an estimate of the average item-construct correlation.

	QD	ED	WPC	RC	IW	DL	PD	SCo	SSu	SC	PR
QD	0.69	0.46 ***	0.66 ***	0.35 ***	−0.16 **	−0.09 *	0.31 ***	−0.02	−0.12 *	−0.01	−0.21 ***
ED		0.72	0.40 ***	0.11 *	−0.01	−0.07	0.32 ***	0.03	0.01	−0.08	−0.12 *
WPC			0.82	0.35 ***	−0.24 ***	−0.16 **	0.14 **	−0.23 ***	−0.18 **	−0.19 ***	−0.29 ***
RC				0.75	−0.31 ***	−0.16 **	−0.16 **	−0.24 ***	−0.24 ***	−0.27 ***	0.39 ***
IW					0.59	0.32 ***	0.45 ***	0.25 ***	0.32 ***	0.46 ***	0.48 ***
DL						0.83	0.19 **	0.20 ***	0.10 *	0.20 ***	0.26 ***
PD							0.65	0.41 ***	0.39 ***	0.43 ***	0.39 ***
SSc								0.80	0.68 ***	0.75 ***	0.39 ***
SSu									0.85	0.44 ***	0.53 ***
SC										0.81	0.46 ***
PR											0.76

*** *p* < 0.001, ** *p* < 0.01, * *p* < 0.05; QD = Quantitative Demands; ED = Emotional Demands; WPC = Work–Privacy Conflict; RC = Role Conflict; IW = Influence at Work, DL = Decision Latitude; PD = possibilities for Development; SCo = Social Support from Colleagues; SSu = Social Support from Supervisor; SC = Sense of Community; PR = Predictability.

**Table 4:** Coefficients in the prediction model in the full sample and comparing the two professional groups.

	Burnout	Job Satisfaction	General Health	Life Satisfaction
**Full Sample (*n* = 466)**				
Quantitative Demands	β = −0.12	β = 0.10	β = 0.03	β = −0.01
Emotional Demands	β = 0.20 ***	β = −0.00	β = 0.05	β = −0.01
Work–Privacy Conflict	β = 0.67 ***	β = −0.22 **	β = 0.09	β = −0.16
Role Conflict	β = 0.10 *	β = −0.12 *	β = −0.04	β = 0.10
Influence at Work	β = −0.09	β = 0.16 *	β = −0.07	β = −0.03
Decision Latitude	β = 0.09	β = −0.05	β = −0.16 **	β = −0.07
Possibilities for Development	β = −0.08	β = 0.34 ***	β = 0.05	β = 0.31 ***
Social Support Colleagues	β = 0.05	β = −0.01	β = 0.12	β = 0.10
Social Support Supervisor	β = −0.05	β = 0.12	β = −0.05	β = 0.01
Sense of Community	β = −0.02	β = 0.16	β = 0.03	β = −0.01
Predictability	β = 0.02	β = 0.16 *	β = 0.16 *	β = −0.06
Burnout			β = 0.62 ***	β = −0.20 ***
Job Satisfaction				β = 0.29 ***
Total Variance Explained	*R*² = 0.54	*R*^2^ = 0.57	*R*^2^ = 0.41	*R*^2^ = 0.41
Moderator analysis comparing the two professional groups (Teachers, *n*_T_ = 227; Social Workers, *n*_SW_ = 239)				
Emotional Demands	β_T_ = 0.35 ***			
	β_SW_ = 0.09 ***			
	∆χ² = 4.34 | *p* = 0.037			
Predictability		β_T_ = −0.13		
		β_SW_ = 0.26 *		
		∆χ² = 4.39 | *p* = 0.036		
Role Conflict			β_T_ = −0.23 *	β_T_ = −0.09
			β_SW_ = 0.07	β_SW_ = 0.19 *
			∆χ² = 4.25 | *p* = 0.039	∆χ² = 4.90 | *p* = 0.027
Influence at Work				β_T_ = 0.19
				β_SW_ = −0.23
				∆χ² = 5.68 | *p* = 0.017
Work–Privacy Conflict				β_T_ = −0.59 ***
				β_SW_ = 0.07
				∆χ² = 5.16 | *p* = 0.023
Quantitative Demands				β_T_ = 0.52 ***
				β_SW_ = −0.08
				∆χ² = 5.56 | *p* = 0.018
Burnout				β_T_ = −0.07
				β_SW_ = −0.36 ***
				∆χ² = 4.07 | *p* = 0.044
Total Variance Explained	*R*²_T_ = 0.59	*R*²_T_ = 0.58	*R*²_T_ = 0.47	*R*²_T_ = 0.55
	*R*²_SW_ = 0.52	*R*²_SW_ = 0.60	*R*²_SW_ = 0.41	*R*²_SW_ = 0.44

* *p* < 0.05; ** *p* < 0.01; *** *p* < 0.001; T = Teachers, SW = Social Workers. Empty cells in the moderator analysis comparing the two professional groups indicate a non-significant value of ∆χ²; coefficients for the full sample can be seen as optimal regardless of sub-groups.

## Data Availability

The datasets generated and analyzed during the current study are not publicly available due to confidentiality but are available on reasonable request from the corresponding author. The set of questionnaires used in this study only included published questionnaires [13,24].
